# Editorial: The mechanism on development and regeneration of inner ear hair cells

**DOI:** 10.3389/fnmol.2022.974270

**Published:** 2022-08-09

**Authors:** Haojie Sun, Binjun Chen, Yu Sun, Hongzhe Li, Dongdong Ren

**Affiliations:** ^1^Department of Otorhinolaryngology, Eye and ENT Hospital, Fudan University, Shanghai, China; ^2^Department of Otorhinolaryngology, Tongji Medical College, Union Hospital, Huazhong University of Science and Technology, Wuhan, China; ^3^VA Loma Linda Healthcare System, Veterans Health Administration, United States Department of Veterans Affairs, Loma Linda, CA, United States

**Keywords:** inner ear, development, regeneration, vestibule, cochlea

The inner ear, which consists of the cochlea and vestibular systems, is a morphologically complex sensory organ. Hair cells (HC), which serve as inner ear sensory cells, are critical for the transduction of mechanical stimulus into auditory and balance signals in the inner ear. After inner ear injury, auditory abnormalities and balance disorders are major consequences. Promoting hair cell regeneration is one of the most promising strategies to address hearing loss and vestibular dysfunction. Exploring the mechanism of inner ear development provides a practical venue to understand the regeneration process of functional hair cells.

Six articles were included in this topic regarding inner ear development. With regard to cochlear development, a pseudo-temporal analysis of single-cell RNA sequencing done by Chen J. et al. focused on the greater epithelial ridge (GER) cells that solely present during cochlear development, and profiled the gene expression landscape of the rat's cochlear basal membrane (P1, P7, P14) to categorize major subtypes of GER cells. The authors documented the trans-differentiation of different GER subtypes to inner (IHC) and outer hair cells (OHC) by trajectory analysis. They also elucidated the key regulatory genes and signaling pathways during trans-differentiation, which is instrumental for future research on HC regeneration. Another study about the organ of Corti (OoC) in the present special topic conducted by Liu et al. dug into the connexin26 (Cx26) protein, which is encoded by the GJB2 gene. The study demonstrated that Cx26 is involved in the maturation of the cytoskeleton during the earlier postnatal development of the OoC. In addition, the polymerization of G-actin into F-actin is prevented in Cx26 knock-down (KD) mice. This study elucidated an underlying mechanism of the OoC deformity caused by Cx26 downregulation. Given that GJB2 mutation is one of the most common etiologies of hereditary deafness, the work is particularly noteworthy.

As to vestibular development, three articles were included in this topic focusing on the development of vestibular HCs and the formation of the vestibular flat epithelium. Chen B.-J. et al. described the role of Rab11a, a small G protein, in the formation of the polarity of cilia, thus revealing that Rab11a is essential to the normal development of cilia, as well as the intraflagellar transport. Another investigation conducted by Yan et al. explored the role of BAIAP2L2, a component of the row 2 complex in stereocilia, which was found to cause degeneration of the mechanotransducing stereocilia in cochlear hair cells when inactivated. Surprisingly, such degeneration was not found in the vestibular HC stereocilia, considering the unaffected morphology, the intact development and function of the vestibule, which might be explained by the differential dependency of CAPZB2 localization on BAIAI2L2 in cochlear and vestibular HCs. Thus, the comparative approach focusing on the phenotypic difference between vestibular and cochlear HC development could provide insights into their development, as well as regeneration.

Degeneration of the vestibular sensory epithelium leads to the formation of flat epithelium (FE) with unclear pathogenesis. He et al. uncovered the role of epithelial-mesenchymal transition (EMT) in this process. Upregulated mesenchymal cell markers in the vestibular FE compared to the normal utricle and robust cell proliferation were observed, which was consistent with GO and DEG analyses following a microarray analysis. The transcriptome features provided by this research provide the foundation for future studies in novel intervention strategies for FE.

Except for HC development, as the first and extremely important synaptic structures formed between IHCs and spiral ganglion neurons (SGN), ribbon synaptic maturity was also included in the topic. The study by Guo et al. found that in the earlier stages of auditory development, under the regulation of autophagy, ribbon synaptic refinement and the pruning of SGN fiber occur and are closely associated with the morphological and functional maturation of ribbon synapses. Further evidence pertaining to IHC functionalities is required to validate the conclusion on inner ear development.

Vestibular and cochlear HCs and SGNs are the major objectives of inner ear regeneration. Based on the past inner ear developmental research, three reviews and original articles were also included in our topic.

To expand the knowledge relating to HC regeneration in adult cochlear explant culture, which is complementary to inner ear organoids to study the inner ear *in vitro*, Li et al. hypothesized that the integral cochlear structure helps maintain the overall inner ear architecture and improve the sensory epithelium survival in culture. To test the hypothesis, they induced trans-differentiation of adult supporting cells to HC-like cells (HCLCs) after HC degeneration by overexpression of Atoh1 in adult mouse cochlear culture with the (surrounding) bone intact. Furthermore, HCLC-neuron connections were observed, proving the feasibility of culturing adult inner ear tissues for future research in regeneration, HC-neuron pathways, and inner ear drug screening.

Regeneration of vestibular HC is critical due to its vulnerability to ototoxic drugs and virus infection and limited restorative capacity after damage. Huang et al. summarized the development of human vestibular hair cells during the entire embryonic stage and the latest research on human vestibular hair cell regeneration. The regenerative potential of human vestibular HC and the application of gene therapy in this field were discussed. The author also pointed out the limitations of current studies and future directions, including some important problems such as how to regenerate and maintain mature and functional HCs, which are imminent issues to resolve in order to propel the field of regeneration of inner ear sensory epithelium, whether in vestibular or cochlear.

Another review about regeneration in our topic is about the SGN, which is a vital component in sensorineural hearing loss. SGNs are the primary neurons that relay sound signals from the inner ear to the brainstem, however, it is vulnerable to noise and ototoxic drugs. Degeneration of SGNs causes irreversible sensorineural hearing loss (SNHL) and cannot be rescued effectively because of their lack of regenerative potential. Wang et al. reviewed the recent advances in research of SGN regeneration including exogenous stem cell transplantation and endogenous glial cell reprogramming. In sum, SGN regeneration is facing a similar challenge to HC regeneration. Considering the progress of synaptic junction formation and the regeneration of hair cells and spiral ganglion neurons, inner ear regeneration is becoming a promising field in otology.

In addition to the development and regeneration of the inner ear, this topic also included a study about the protective effect of shikonin, a pigment isolated from traditional Chinese herbal medicine, on spiral ganglion cells. Du et al. found that shikonin reduced the ouabain-induced auditory nerve damage and increase the expression of Nrf2 and its downstream molecules HO-1 and NQO1. Shikonin treatment enhanced the antioxidant capacity of SGNs and spiral ganglion Schwann cells, promoted cell proliferation, and inhibited apoptosis by activating the Nrf2/antioxidant response elements signal pathway, thereby testifying it as a candidate therapeutic drug for neurological deafness.

Overall, this topic, with ten papers encompassing various subjects in the area of inner ear development, revealed the promising potential of this research interest and promoted the progress of this lesser-known research direction.

## Author contributions

DR, HS, and HL designed the literature. HS and BC co-authored the manuscript and reviewed previous research. All authors contributed to the article and approved the submitted version.

## Conflict of interest

The authors declare that the research was conducted in the absence of any commercial or financial relationships that could be construed as a potential conflict of interest.

## Publisher's note

All claims expressed in this article are solely those of the authors and do not necessarily represent those of their affiliated organizations, or those of the publisher, the editors and the reviewers. Any product that may be evaluated in this article, or claim that may be made by its manufacturer, is not guaranteed or endorsed by the publisher.

